# Frequency Domain Template Subtraction Approach to Attenuate Maternal Electrocardiogram in Fetal Electrocardiogram

**DOI:** 10.3390/neurosci5020013

**Published:** 2024-05-25

**Authors:** Susan Wang, Pooneh Roshanitabrizi, Anita Krishnan, R. B. Govindan

**Affiliations:** 1 Division of Cardiology, Children’s National Hospital, Washington, DC 20010, USA; swang@childrensnational.org (S.W.); akrishna@childrensnational.org (A.K.); 2 Sheikh Zayed Institute for Pediatric Surgical Innovation, Children’s National Hospital, Washington, DC 20010, USA; proshnani2@childrensnational.org; 3 Department of Pediatrics, The George Washington University School of Medicine, Washington, DC 20052, USA; 4 Prenatal Pediatrics Institute, Children’s National Hospital, Washington, DC 20010, USA

**Keywords:** spectral coherence, transfer function gain, time domain filter

## Abstract

We develop a frequency domain template subtraction approach to attenuate the maternal ECG in the abdominal ECG measured from pregnant women. The proposed approach was tested on five public fetal ECG datasets simultaneously measured with ECG from the fetal scalp. The method’s performance was compared with the template subtraction approach in the time domain using the accuracy and association metrics. The accuracy was calculated by counting the number of fetal complexes in the processed data that coincided with the fetal complexes in the scalp fetal ECG. The association is quantified as the coherence between the processed data and the gold standard. The maximum coherence values calculated for each approach were compared using the paired *t*-test. Our results showed no difference in the accuracy between the frequency and time domain approach (*p* = 0.733). However, the association was higher between the frequency domain data and the gold standard compared to the template subtraction data and the gold standard (*p* = 0.049), indicating that the frequency domain approach yielded a signal that resembled that of the scalp ECG compared to the time domain approach.

## 1. Introduction

Fetal electrocardiogram (FECG) is the only continuous fetal signal we can collect from a pregnant mother by placing electrodes on the abdomen. The foundation for FECG research comes from the pioneering studies of Hon [1], and these studies showed an association between fetal distress during the intrapartum period and heart rate calculated using the scalp FECG [1,2]. Over the past two decades, several commercial devices have been developed to monitor FECG [3,4,5,6]. In addition to the low-cost FECG technologies, superconducting quantum interference device-based fetal magnetocardiography technologies have also been developed [7]. In all these technologies, the maternal ECG (MECG) is a major interference in FECG. Several signal processing techniques have been developed to attenuate the interfering MECG, and they include projection operators [8,9], principal component analysis [10], independent component analysis, and frequency domain or coherence subtraction approach [10,11]. Our recent work demonstrated that coherence subtraction approach outperformed other approaches [10]. The coherent subtraction approach requires an independently recorded MECG as a reference signal to attenuate the maternal cardiac cycles in the abdominal ECG. In studies where MECG was not recorded simultaneously with abdominal ECG, coherent subtraction approach cannot be applied. To address this limitation, in this work, we develop a frequency domain template subtraction approach to attenuate MECG in FECG. We apply our method to five FECGs measured from the abdomen of the pregnant women simultaneously with fetal scalp ECG and show that our method attenuates the MECG on par with the template subtraction approach. However, the quality of the FECG obtained from the frequency domain template subtraction is superior to the one obtained using the template subtraction approach.

## 2. Materials and Methods

We used the fetal scalp ECG signal measured simultaneously with four channels of FECG from the maternal abdomen and made available in the open repository [12,13,14]. ECGs were measured using Ag-AgCl (3 M Red Dot 2271) electrodes. The pregnancy was singleton in all cases. The abdominal skin was cleaned using an abrasive material to enhance the conductance. The signals were bandpass filtered between 1 and 150 Hz and digitized at 16-bit resolution. The sampling rate was 1000 Hz. A 401-millisecond (200 milliseconds on either side of every sample) median filter was calculated for abdominal ECGs, and the running median was subtracted from the abdominal ECGs to attenuate low-frequency artifacts such as baseline drifts. The maternal R-waves were identified using the abdominal ECG for each channel using the Pan-Thompkins approach [15]. The maternal ECG was attenuated using the template subtraction and frequency domain template subtraction approaches. Since we downloaded these data from a public repository, we did not obtain institutional review board approval or consent from the participants to use the data.

### 2.1. Template Subtraction

A generic algorithm for the template subtraction approach is as follows. The template subtraction approach is applied to every (maternal) R-wave, by selecting x milliseconds of samples before and y milliseconds of samples after the selected R-wave, and forming a cardiac vector. This vector is constructed for every cardiac cycle, and finally, all the vectors are averaged to obtain a template. Then, the approach revisits every cardiac cycle and fits the cardiac cycle and the template using a least-square approach. To this end, the approach predicts the cardiac cycle using the least-square coefficients, and the predicted cardiac cycle is subtracted from the actual cycle. The cardiac cycle at this instant is replaced with the yielded residue. This procedure is repeated for every cardiac cycle in the record. In this work, we used x as 200 and y as 300. For details of the approach used in this work, we refer to a previous work by Behar et al. [16].

### 2.2. Frequency Domain Template Subtraction

This approach follows the same procedure as in the template subtraction approach until the template construction part. The template is transformed into frequency domain using the Fourier transform $\left(F_{T}(\omega \right)$). Then, the approach revisits every cardiac cycle and transforms the cardiac cycle at the ‘i-th’ instance into the Fourier domain $\left(F_{i}\left(\omega \right)\right)$. Coherence spectrum is calculated using the Fourier coefficients as follows:

$$C\left(\omega \right)=\frac{{\left\lfloor \sum_{i=1}^{N}F_{T}\left(\omega \right)\cdot \;F_{i}{\left(\omega \right)}^{†}\right\rfloor }^{2}}{\sum_{i=1}^{N}F_{T}\left(\omega \right)\cdot F_{T}{\left(\omega \right)}^{†}\cdot \sum_{i=1}^{N}F_{i}\left(\omega \right)\cdot F_{i}{\left(\omega \right)}^{†}}\;,$$

where † indicates the complex conjugate operator with N being the number of cardiac cycles. Notably, in the above formula, $\sum_{i=1}^{N}F_{T}\left(\omega \right)\cdot F_{T}{\left(\omega \right)}^{†}$ is equal to $N\cdot {\left|F_{T}\left(\omega \right)\right|}^{2},$ with $\left|\cdot \right|$ being the absolute operator. The numerator in the coherence spectrum indicates the estimate of the cross-spectrum spectrum between the template and the cardiac cycles in the record, and the denominator indicates the product of the auto-spectra of the template and the cardiac cycles in the record. Following [10], the spectral gain is defined as follows:

$$G\left(\omega \right)=\;\frac{\sum_{i=1}^{N}F_{T}\left(\omega \right)\cdot \;F_{i}{\left(\omega \right)}^{†}}{\sum_{i=1}^{N}F_{T}\left(\omega \right)\cdot F_{T}{\left(\omega \right)}^{†}}$$

The subtraction of the template from the cardiac cycles at instance i is given as follows:

$$R_{i}\left(w\right)=F_{i}\left(\omega \right)-F_{T}\left(\omega \right)*G{\left(\omega \right)}^{†},$$

where $R_{i}\left(\omega \right)$ is the residue, which is then transformed back to the time domain by taking the inverse Fourier transform. The cardiac cycle at instant i is replaced with the transformed residue $R_{i}\left(\omega \right).$ This procedure is repeated for all cardiac cycles in the record.

### 2.3. Performance Assessment and Statistical Considerations

To assess the performance of each approach, we displayed their outputs alongside the abdominal ECG and the scalp ECG. A pediatrician (SW) initially trained by a pediatric cardiologist (AK) visually reviewed those tracings and counted: (1) the number of fetal cardiac cycles yielded by each approach that coincided with the cardiac cycles in the scalp ECG and (2) the number of poorly attenuated maternal cycles yielded by each approach. The proportion of cardiac cycles identified by each approach was calculated as the ratio of the total counts of cardiac cycles that coincided with those in the scalp ECG to the total number of cardiac cycles in the scalp ECG. Similarly, the proportion of the poorly attenuated maternal cycles was calculated as the ratio of the total counts of poorly attenuated maternal cardiac cycles yielded by each approach to the total number of maternal cycles in the abdominal ECG. These two proportions were compared between the two approaches using the Chi-square proportionality test. The quality of each approach’s output was assessed by calculating the coherence between the output of each approach and the fetal scalp ECG. The frequency resolution of the coherence was 0.1 Hz. The output that yields high coherence indicates the FECG yielded by that approach closely resembles that of the fetal scalp ECG. The maximum coherence was calculated from the coherence spectra and compared between the two approaches using the paired *t*-test. In all comparisons, *p* < 0.05 was considered statistically significant. The α-level confidence limit for coherence was calculated using $1-{\left(1-\alpha \right)}^{\frac{1}{M-1}}$, with M being the number of epochs used in the spectral estimation, which is 30 in our case. α was set to 0.999 in our calculations.

## 3. Results

The database comprised five subjects. All five women were in labor, and their gestational ages ranged between 38 and 41 weeks. For each subject, there are five channels, four abdominal ECG channels, and one fetal scalp ECG channel. Both template subtraction and frequency domain template subtraction were applied to every abdominal channel. The channel that yielded best results, demonstrating higher agreement with the fetal scalp ECG, was selected for further analysis.

**Figure 1:**
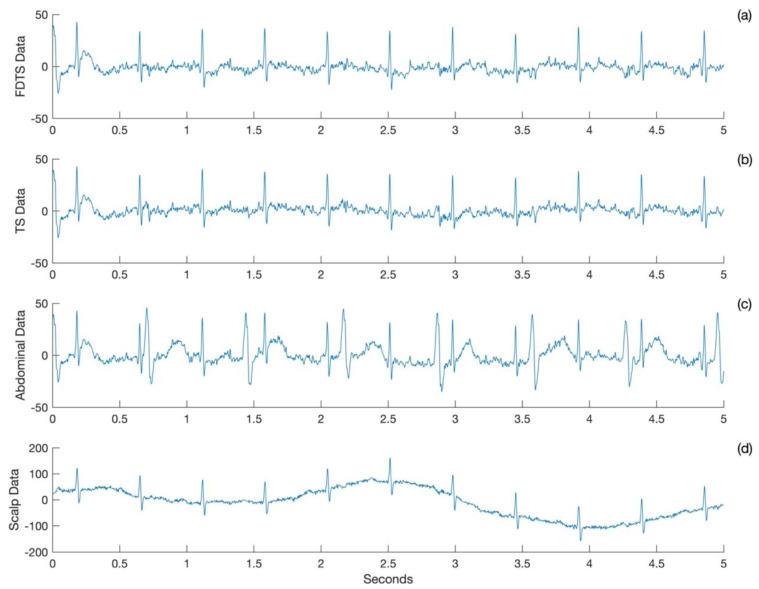
Sample ECG acquired for Subject 1. Output of (**a**) frequency domain template subtraction (FDTS) and (**b**) template subtraction (TS) approaches. (**c**) Abdominal ECG and (**d**) scalp ECG.

A plot of five seconds of FECG was obtained from the frequency domain template subtraction approach (Figure 1a), template subtraction approach (Figure 1b), abdominal ECG (Figure 1c), and scalp ECG (Figure 1d) for Subject 1. The FECG obtained from the two approaches matches with the scalp ECG, and both of them have attenuated the maternal cardiac signals to the baseline.

**Figure 2:**
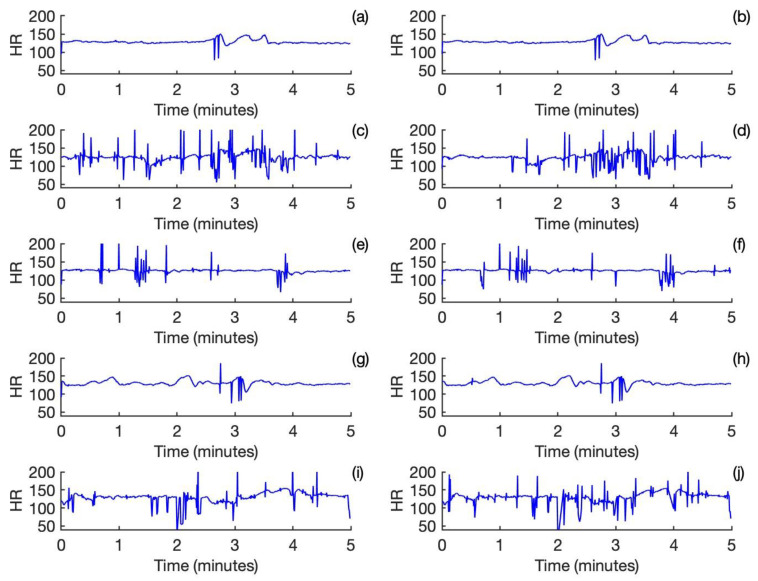
Fetal heart rate calculated from the outputs of the frequency domain template subtraction approach for Subjects 1-5 is shown in (**a**,**c**,**e**,**g**,**i**). Fetal heart rate calculated from the outputs of the template subtraction approach for Subjects 1-5 is shown in (**b**,**d**,**f**,**h**,**j**).

The fetal heart rates calculated for the output of the frequency domain template subtraction approach are shown in Figure 2a,c,e,g,i. Correspondingly, the fetal heart rates calculated for the output of the template subtraction approach are shown in Figure 2b,d,f,h,j. For Subject and Subject 4, there is a nearly perfect agreement between the two approaches. For Subject 2 and Subject 3, which are noisy, the performance is relatively poor. Both approaches have performed reasonably well for Subject 5, despite it being partly noisy.

**Figure 3:**
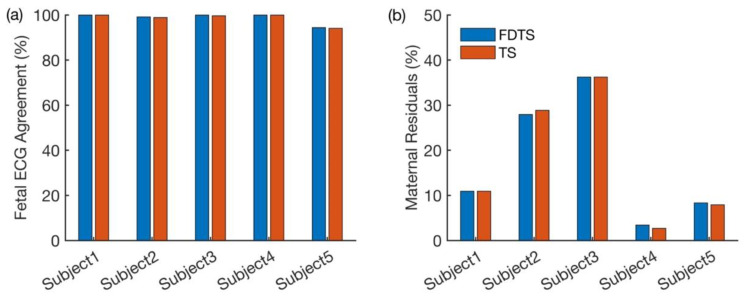
Comparison of performances of both approaches. (**a**) Proportions of cardiac cycles identified by both approaches coincided with those in the fetal scalp ECG. (**b**) Proportions of the maternal residual observed in the outputs of both approaches.

Figure 3a shows the proportions of fetal cardiac cycles obtained from each approach that coincided with the cardiac cycles in the fetal scalp ECG. Figure 3b shows the percentage of the maternal residuals yielded by both approaches for each subject. From Figure 3a, it is evident that both approaches have performed well. In Figure 3b, higher proportions of maternal residuals are observed in Subjects 2 and 3, possibly due to noisy signals. Even in this comparison, both approaches yielded consistent results. The *p*-values obtained from the Chi-square proportionality test are shown in Table 1.

**Figure 4:**
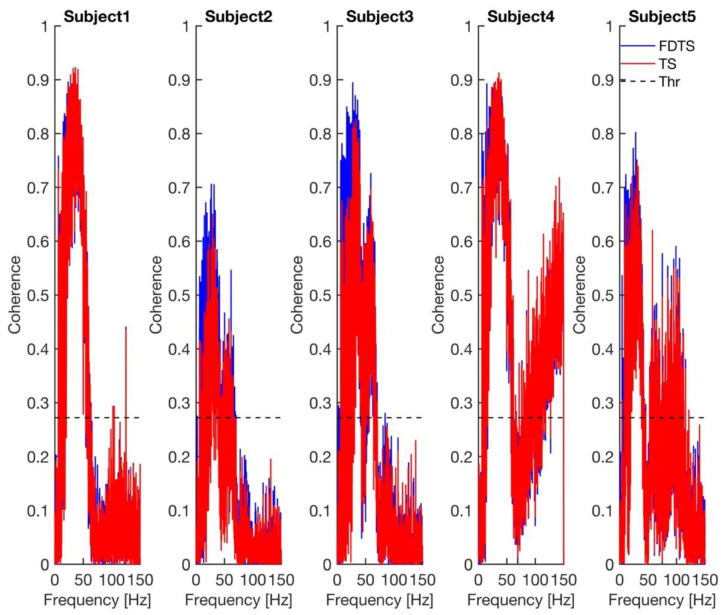
Coherence between each approach’s output and scalp ECG for every subject. The dashed line in all plots represents the 99.99% confidence limit for coherence. FDTS, TS, and Thr denote frequency domain subtraction, template subtraction, and coherence threshold, respectively.

The *p*-values in Table 1 show that both approaches have performed equally well. The coherence spectra calculated between the approaches’ output and scalp ECG for every subject are shown in Figure 4. The coherence spectra obtained for the frequency domain template subtraction approach’s output and scalp ECG were higher than those of the template subtraction approach’s output and scalp ECG in subjects 2, 3, and 5. The *p*-value obtained from the paired *t*-test, comparing the maximum coherence between the two approaches, is 0.049. The mean (standard deviation) maximum coherence values of the frequency domain template subtraction and template subtraction are 0.84 (0.09) and 0.81 (0.12), respectively. This indicates that coherence values obtained for the frequency domain template subtraction approach are higher than the template subtraction approach (see Figure 4).

## 4. Discussion

We describe a frequency domain template subtraction approach to attenuate maternal cardiac cycles in the abdominal ECG measured from pregnant women. The method tested on the five datasets shows performance statistically similar to its time domain equivalence. Notably, the FECG obtained using the frequency domain template subtraction approach closely resembles the scalp ECG, making frequency domain template subtraction a preferable method over its time domain counterpart.

A fetal cardiogram is an excellent antepartum surveillance tool. Most commercial FECG systems are designed for intrapartum monitoring [3]. However, their applications have been extended to the antenatal period. The major challenge in FECG research is the separation of the huge maternal cardiac interference from the FECG [17]. Some commonly used approaches, such as independent component analysis (ICA) or projection operators, require many channels to reliably identify the maternal source signals, which will then be attenuated in the subsequent steps [9,18]. The introduced frequency domain approach recently requires reference maternal cardiac signal measured from an independent source to attenuate the maternal cardiac cycles in the abdominal ECG [10].

Similarly, Weiner filter [10], Kalman filter [19], and its extension have been used to attenuate the maternal cardiac cycles in the abdominal ECG. Techniques such as the Kalman filter and its extension, Weiner filter, and template subtraction will work well for stationary data. However, several physiological processes, such as fetal movements, the mother’s respiration, and the mother’s uterine contraction, can make FECG non-stationary. A method that is robust against non-stationarity can reliably attenuate the maternal ECG. Our data indicate that the frequency domain template subtraction approach might be more robust to the non-stationarity than the template subtraction approach.

For most parts, the template subtraction works reasonably well, but in those instances where the maternal cardiac cycle overlaps with the fetal cardiac cycle, it may suffer. The frequency domain template subtraction, since it works in the frequency domain, is able to perform better than its time domain counterpart. In the datasets that we used in this work, the fetal movement was not observed, and the heart rate was pretty constant throughout the recording averaging around 130 bpm. However, during the antenatal period, the fetal heart rate can go up to 190 bpm during the active awake state [8]. This increased rate may lead to more overlapping instances of maternal and fetal cardiac cycles. Additionally, in the datasets used, the absence of amniotic fluid in the uterus resulted in a slightly higher FECG amplitude compared to typical antenatal recordings. However, during the antenatal period, there will be contributions from the amniotic fluid, which can dampen the amplitude of the FECG. The real test of the time domain template subtraction approach will be its application to attenuate the maternal ECG in the abdominal ECG collected during the antenatal period. In our earlier work, we demonstrated the superior performance of the frequency domain subtraction approach compared to the template subtraction approach [10].

Our method shares limitations with the template subtraction approach. These approaches highly rely on the accurate detection of maternal cardiac complexes. The detection of the maternal cardiac cycle will fail when there is a drift in the baseline. The approaches will not attenuate the maternal cardiac cycles in those instances. Additionally, the non-stationarity introduced by the mother’s uterine contractions can impact the performance of these approaches.

In conclusion, we have developed a frequency domain template subtraction method and demonstrated its slightly superior performance compared to its time domain counterpart for non-stationary data. These methods hold potential applications in FECG research for attenuating maternal cardiac signals, particularly when no reference maternal signal is available.

## Figures and Tables

**Table 1:** Comparison of the proportions of fetal cardiac cycles obtained from each approach with those in fetal scalp ECG using the Chi-square test. Also, the comparison of the proportions of maternal cardiac cycles observed in each approach’s output using the Chi-square test is given in this table.

Comparison	*p*-Values				
	Subject 1	Subject 2	Subject 3	Subject 4	Subject 5
Processed ECG vs. scalp ECG	1	0.56	1	1	0.81
Maternal ECG residuals	1	0.76	1	0.54	0.81

## Data Availability

The data analyzed in this study were downloaded from the following open repository: https://physionet.org/content/adfecgdb/1.0.0/ 27 July 2022.
